# Supplementary material for: Coarse-Grained Simulations on Polyethylene Crystal Network Formation and Microstructure Analysis
Source: Polymers (Basel). 2024 Apr 7;16(7):1007. doi: 10.3390/polym16071007 (PMC11013834; doi:10.3390/polym16071007)
Supplement: Supplementary file 1 [file polymers-16-01007-s001.zip › polymers-2924181-supplementary.pdf]

Supporting Information

Coarse-Grained Simulations on Polyethylene Crystal Network Formation and Microstructure Analysis

Mohammed Althaf Hussain[1,*], Takashi Yamamoto[2], Syed Farooq Adil[3], and Shigeru Yao[1,*]

[1] Central Research Institute, Fukuoka University, 8-19-1 Nanakuma, Jonan-Ku, Fukuoka 814-0180, Japan
[2] Graduate School of Science and Engineering, Yamaguchi University, Yamaguchi 753-8512, Japan
[3] Department of Chemistry, College of Science, King Saud University, P.O. Box 2455, Riyadh 11451, Saudi Arabia; sfadil@ksu.edu.sa

*Correspondence: altaf.mh7@gmail.com; shyao@fukuoka-u.ac.jp

Content	Page No.
**Table S1.** The density and box dimensions of the $10C_{1000}$ model at each stage of the MD simulations using PYS/R forcefield.	S2
**Figure S1.** Potential Energy decomposition of isotropically melting amorphous state at 450K and 1 atm pressure.	S3
**Figure S2.** Amorphous state S-S curves for the $10C_{1000}$ model at 300K and zero pressure conditions using the NPT ensemble. Each model deformed to 500% of the initial box length of the simulation box's X, Y, and Z directions.	S4

Table S1. The density and box dimensions of the $10C_{1000}$ model at each stage of the MD simulations using PYS/R forcefield.

Conditions	$10C_{1000}$	
	ρ	Cell
**Data file and NVT melting at 450K**	*0.931*	a = 50.0
		b = 50.0
		c = 100.0
**NPT Equilibration model at 450K**	*0.769*	a = 53.282
		b = 53.282
		c = 106.564
**Quenched model from 450K to 300K**	*0.856*	a = 51.405
		b = 51.405
		c = 102.81
**Isothermally cooled at 300K**	*0.913*	a = 50.313
		b = 50.313
		c = 100.626

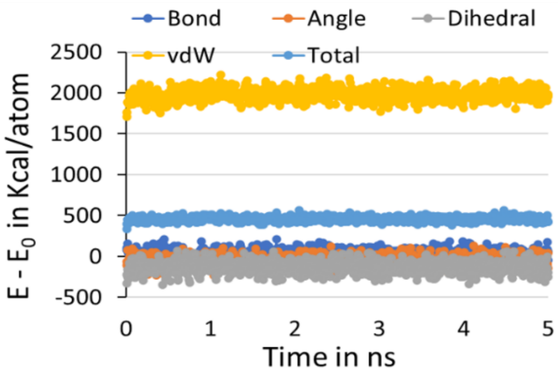

Figure S1. Potential energy decomposition of isotropically melting amorphous state at 450K and 1 atm pressure.

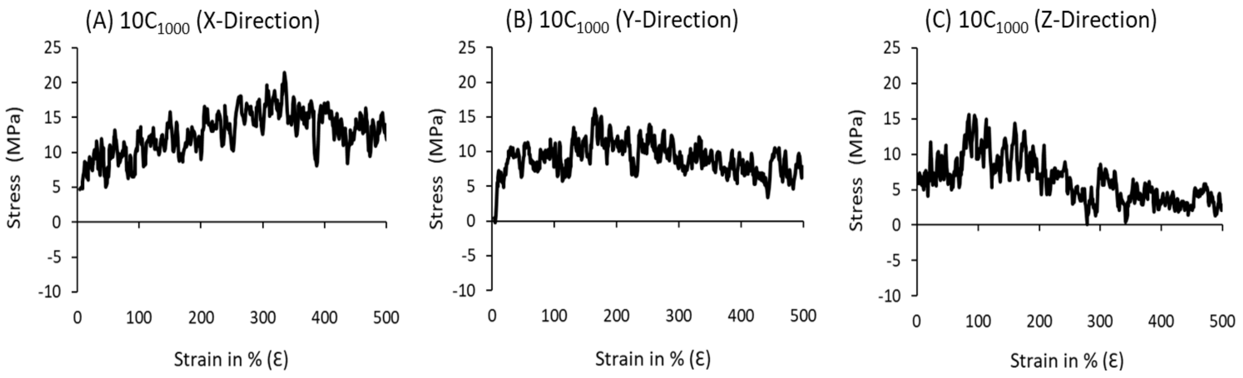

Figure S2. Amorphous state S-S curves for the $10C_{1000}$ model at 300K and zero pressure conditions using the NPT ensemble. Each model deformed to 500% of the initial box length of the simulation box's X, Y, and Z directions.
